# MALT lymphoma of the left biceps muscle: a rare case with an interesting presentation

**DOI:** 10.1186/s40064-016-2880-3

**Published:** 2016-07-28

**Authors:** Ipek Yonal-Hindilerden, Fehmi Hindilerden, Ibrahim Oner Dogan, Meliha Nalcaci

**Affiliations:** 1 Division of Hematology, Department of Internal Medicine, Istanbul Medical Faculty, Istanbul University, Istanbul, Turkey; 2 Hematology Clinic, Istanbul Bakırkoy Sadi Konuk Training and Research Hospital, Istanbul, Turkey; 3 Department of Pathology, Istanbul Medical Faculty, Istanbul University, Istanbul, Turkey

**Keywords:** Extranodal marginal zone lymphoma (MZL), Mucosa-associated lymphoid tissue (MALT), Non-Hodgkin lymphomas (NHLs), Skeletal muscle

## Abstract

**Background:**

Extranodal marginal zone lymphoma (MZL), also called mucosa-associated lymphoid tissue (MALT) lymphoma accounts for 7–8 % of non-Hodgkin lymphomas (NHLs) and most commonly involves the stomach. However, muscle involvement is very rare.

**Case description:**

A 57-year-old woman was referred to our orthopaedics and traumatology clinic with a painful lump in the left arm. Physical examination revealed a red-colored mass on the left arm and an enlarged lymph node measuring almost 5 cm in the left axillary region and 3 cm in the right axillary region. Tru-cut biopsy of the mass in the left arm was consistent with MZL. The diagnosis was MALT lymphoma infiltrating the skeletal muscle (stage IIEA). R-CHOP was started. Two additional infusions of rituximab were administered after the sixth cycle of R-CHOP. Then, the patient received radiotherapy to the left arm at a dose of 30 Gy. After 1 year of follow-up, the patient had no evidence of disease.

**Discussion and evaluation:**

MALT lymphoma arises in a number of epithelial tissues. The clinical presentation of MALT lymphoma varies depending upon the tissue involved. To our knowledge, rare cases of MALT lymphoma of the skeletal muscle have been reported. Although the available literature suggests that primary skeletal muscle NHL with advanced stage is associated with poor prognosis, the case presented here suggests that rituximab based combination therapy followed by radiotherapy can be an effective treatment for primary skeletal MALT lymphoma.

**Conclusion:**

There is limited data regarding the prognosis and treatment of MALT lymphoma of the skeletal muscle. This case implies that rituximab based combination therapy followed by radiotherapy should be considered for the treatment of primary skeletal MALT lymphoma.

## Background

Extranodal marginal zone lymphoma (MZL), also called mucosa-associated lymphoid tissue (MALT) lymphoma accounts for 7–8 % of non-Hodgkin lymphomas (NHLs) and most commonly involves the stomach (Cavalli et al. 2001). The most common manifestation sites of nongastric MALT lymphomas include the salivary glands, Waldeyer’s ring, the thyroid, the upper airways, the lung, the ocular adnexa, the breast, the liver, the urothelial system, the skin, the dura and other soft tissues (Anderson et al. 1997; Joshi et al. 2012). Involvement of muscle is very rare and constitutes <1 % of all MALT lymphomas (Zucca et al. 2003). Herein, we present an adult diagnosed with MALT lymphoma of the left biceps muscle with enlarged distant lymph nodes, who was treated with combined chemo-immunotherapy and radiotherapy and remains disease free after 1 year of follow up.

## Case description

**Fig. 1:**
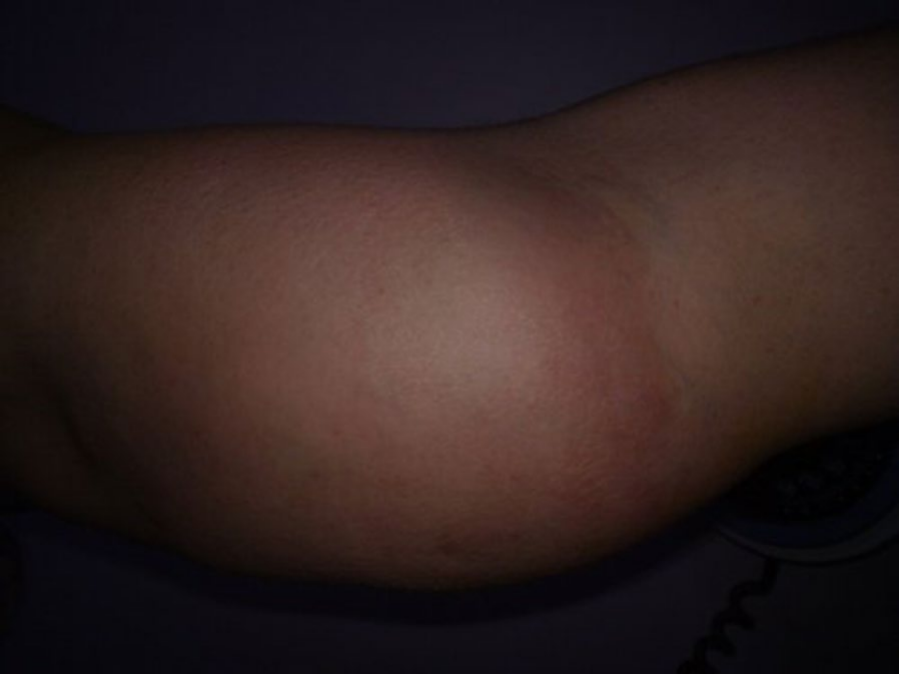
The appearance of mass and skin erythema at the left arm

**Fig. 2:**
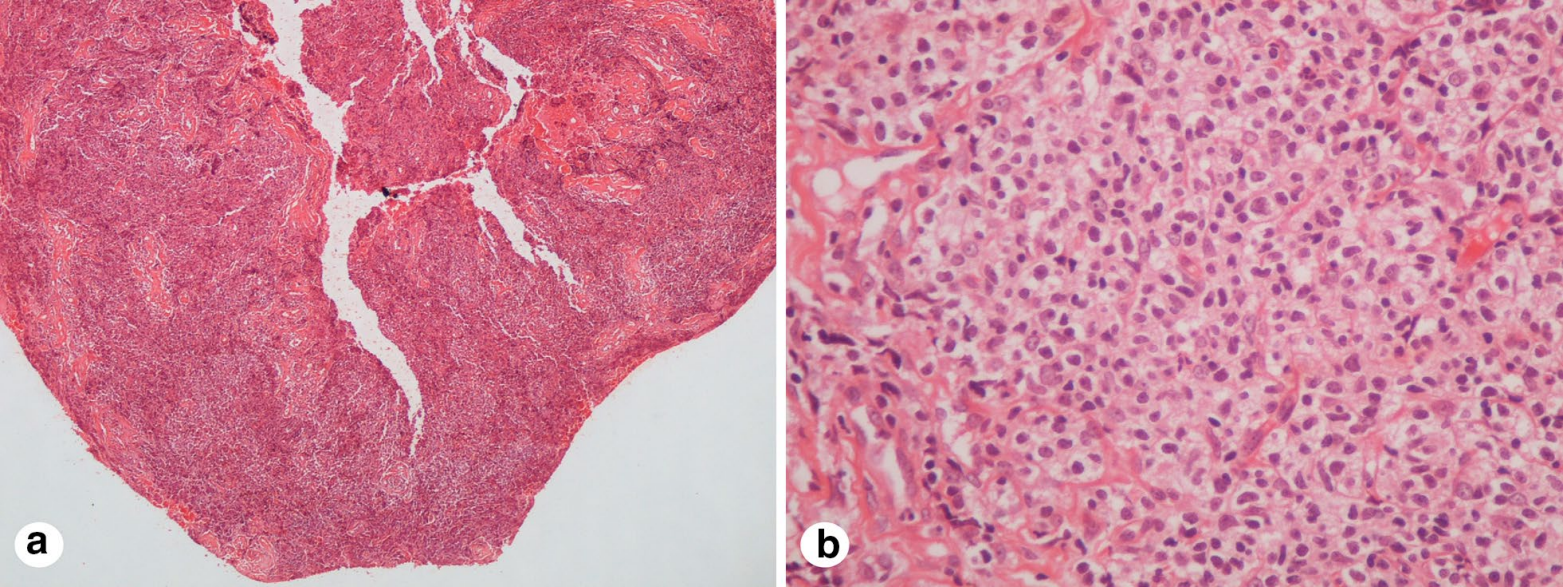
Tru-cut biopsy of the mass in the left arm revealed infiltration of atypical centrocyte-like cells with or without clear cytoplasm (**a**; ×40), (**b**; ×400)

**Fig. 3:**
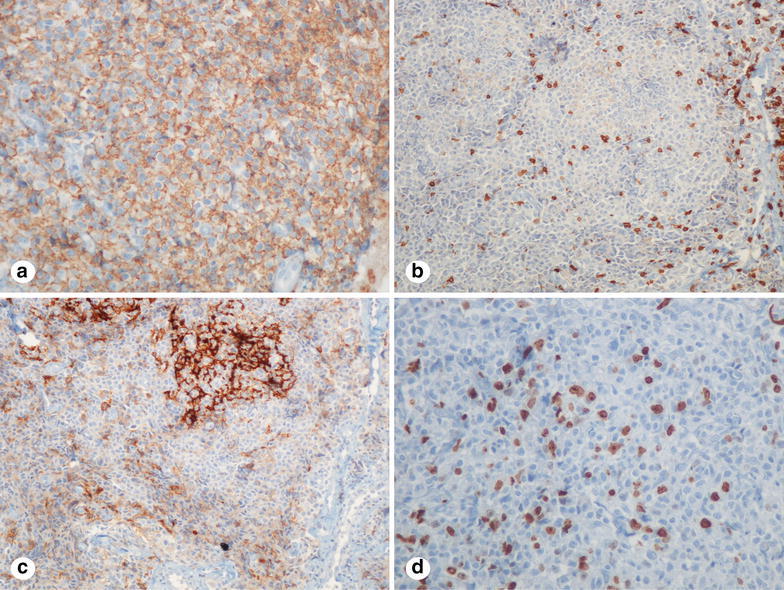
Immunohistochemical features of the infiltrated cells in the left arm expressed CD20 (**a**; ×200), stained negative for CD5 (**b**; ×100), stained positive for CD21 in follicular dendritic cell network (**c**; ×100), 30 % positivity for Ki-67 (**d**; ×200)

A 57-year-old woman was referred to our orthopaedics and traumatology clinic with a painful lump in the left arm. Physical examination revealed a red-colored mass on the left arm (Fig. 1) and an enlarged lymph node measuring almost 5 cm in the left axillary region and 3 cm in the right axillary region. Magnetic resonance imaging (MRI) of the left arm showed a 17.7 × 5.8 × 7.3 cm enhancing mass in the medial aspect of the left biceps muscle and multiple left axillary lymph nodes with a maximal diameter of 4.8 × 3.4 cm. Tru-cut biopsy of the mass in the left arm revealed infiltration of atypical centrocyte-like cells with or without clear cytoplasm with a Kİ-67 proliferative index of 30 % and stained positive for CD20 and pax-5 and negative for CD5, CD10 and bcl-2, positive for CD21 in the follicular dendritic cell network, findings consistent with MZL (Figs. 2, 3). With a final diagnosis of MALT lymphoma infiltrating the skeletal muscle, she was referred to our hematology department. The patient had no B symptoms. Her hemoglobin was 12 g/dL, white blood cell count 8420/mm^3^ with a lymphocyte count 2800/mm^3^ and platelet count was 292,000/mm^3^. On biochemical tests, the following were abnormal: lactate dehydrogenase 1218 U/L (240-480), CRP 40 mg/L (0–5), ESR 120 mm/h (0–20) and β_2_-microglobulin level 3.52 mg/L (0.7–1.8). Serum protein electrophoresis showed polyclonal gammopathy with an increased component with gamma fraction of 1.51 g/dL. Hepatitis B, C and HIV infection serology and screening for autoimmune disorders were negative. Bone marrow biopsy was negative. Cervical computed tomography (CT), and abdominopelvic CT were normal. On chest CT, there were multiple left axillary lymph nodes with a maximal diameter of 5 cm and a 3 cm right axillary lymph node. Diagnosed with stage 2 disease, R-CHOP (rituximab, cyclophosphamide, doxorubicin, vincristine, methylprednisolone) was initiated. After 6 cycles, mass in the medial aspect of the left biceps muscle regressed to a size of 3.2 × 1.1 × 1 cm and bilateral axillary lymph nodes totally disappeared. Two additional infusions of rituximab were administered after the sixth cycle of R-CHOP. Subsequently, the patient underwent radiotherapy to the left arm at a dose of 30 Gy. Physical examination 1 and 6 months and 1 year after treatment revealed no palpable residual mass, swelling or skin erythema. 6-months and 1-year after completion of treatment, MRI of the left humerus demonstrated no evidence of residual disease. Also, cervical, chest and abdominopelvic CT at 1 year showed no disease involvement.

## Discussion and evaluation

MALT lymphoma arises in a number of epithelial tissues, including the stomach, salivary gland, lung, small bowel and thyroid. MALT lymphoma constitutes about 5 % of all NHLs and almost 50 % of all gastric lymphomas (Armitage and Weisenburger 1998). Persistent antigenic stimulation due to autoimmune processes or chronic infectious conditions, such as H pylori gastritis, Sjögren syndrome and Hashimoto thyroiditis predispose to development of MALT (Isaacson and Du 2004). Data from epidemiological and molecular studies favor a multistage theory (Zucca et al. 1998; Seydel et al. 2003). In gastric MALT lymphomas which have been extensively studied, it is considered that the molecular mechanism involves the stimulation of antigen receptor by autoantigen and co-stimulatory molecule CD40 by H pylori-specific T cells. MALT lymphoma develops as marginal zone memory B cells undergo somatic mutation and efface the normal B cell population (Hamoudi et al. 2010).

The clinical presentation of extranodal MZL varies depending upon the tissue involved. Stomach is the most common site of involvement (Cavalli et al. 2001). Involved field radiation therapy for treatment of stomach MALT has been well established, yielding excellent outcomes with over 90 % local control reported in most studies (Tsang et al. 2001; Tsai et al. 2007). Treatment of nongastric MALT lymphomas is much less defined in the literature compared to gastric MALT lymphomas. To our knowledge, rare cases of MALT lymphoma of the skeletal muscle have been reported (Bozzola et al. 2005; Gill et al. 2006; Edwards-Bennett et al. 2011). The involvement sites of previously reported MALT lymphomas of skeletal muscle include the distal biceps and proximal triceps muscles in one case, right triceps muscle in one case and quadriceps muscle in another case (Bozzola et al. 2005; Gill et al. 2006; Edwards-Bennett et al. 2011). One published case presented with stage IV disease with pulmonary involvement. That patient was treated initially with 6 cycles of CHOP and with radiation therapy for local relapse 4 years later, and finally with chemotherapy for a second relapse (Gill et al. 2006). The aforementioned case suggests that primary skeletal lymphoma shows more aggressive behavior, presents as advanced stage, and is associated with poor outcome (Gill et al. 2006). On the other hand, one previous case presented with early stage (IE) and was treated with involved field radiation therapy resulting in complete response (Edwards-Bennett et al. 2011). The available data is very limited and probably suggests that primary skeletal muscle NHL with advanced stage is associated with poor prognosis, the case presented here suggests that rituximab based combination therapy followed by radiotherapy can be an effective option for treatment of primary skeletal MALT lymphoma. Yet, it needs to be mentioned that 1 year-follow up is too short to support the long term effectiveness of this approach for an indolent lymphoma.

## Conclusion

In conclusion, a 57-year-old woman with MALT lymphoma infiltrating the skeletal muscle was successfully managed by 6 cycles of R-CHOP plus two additional infusions of rituximab and radiotherapy. There is limited reported data regarding the prognosis and treatment of MALT lymphoma of the skeletal muscle. We conclude that rituximab based combination therapy and subsequent radiotherapy can be an effective treatment for primary skeletal MALT lymphoma.

## Abbreviations

MZL: extranodal marginal zone lymphoma
MALT: mucosa-associated lymphoid tissue
NHLs: non-Hodgkin lymphomas
MRI: magnetic resonance imaging
